# A Comparative Study of Automated Pulsed Bolus versus Continuous Basal Infusion on Distribution of Contrast in the Transversus Abdominis Fascial Plane in a Cadaver: A Technical Report

**DOI:** 10.7759/cureus.5664

**Published:** 2019-09-16

**Authors:** Elird Bojaxhi, Steven R Clendenen

**Affiliations:** 1 Anesthesiology, Mayo Clinic, Jacksonville, USA

**Keywords:** : transversus abdominis plane (tap) block, continuous nerve catheter, intermittent pulse dosing

## Abstract

Integrating regional anesthesia and multi-modal pain management is a well described and successful strategy to reduce post-operative pain. The use of transversus abdominis plane (TAP) blocks has been well-described for abdominal surgery, which includes various injection sites to improve analgesic coverage and catheter usage to prolong duration of analgesia. After a cadaver contrast study, our investigation illustrates that, for a TAP catheter block, a programmed intermittent bolus provides greater spread of the injection in the fascial plane as compared to a continuous infusion. Clinical trials are needed to investigate if these findings translate to greater analgesic coverage of the anterior abdominal wall, particularly in the subcostal region.

## Introduction

Transversus abdominis plane (TAP) blocks for abdominal surgery have been shown to decrease immediate postoperative pain scores and reduce opioid requirements for the first 24 hours [1]. The duration of the TAP block can be prolonged by the use of peripheral nerve catheters, and this practice is replacing the use of epidural catheters for abdominal surgery [2]. In order to provide effective analgesia after abdominal surgery, the block needs to cover multiple thoracolumbar nerves innervating the abdominal wall potentially from T6 to L2 [3]. Therefore, spread of local anesthetic further along the fascial plane of the TAP block may provide an improved analgesic efficacy. Recently, local anesthetic delivery systems with the ability to deliver the infusion as a programmed intermittent bolus (PIB) or as a continuous infusion (CI) have been developed [4]. The goal of this cadaveric study is to investigate the spread of equal volume of contrast in the transversus abdominis fascial plane between PIB and CI modes of delivery.

## Technical report

**Figure 1:**
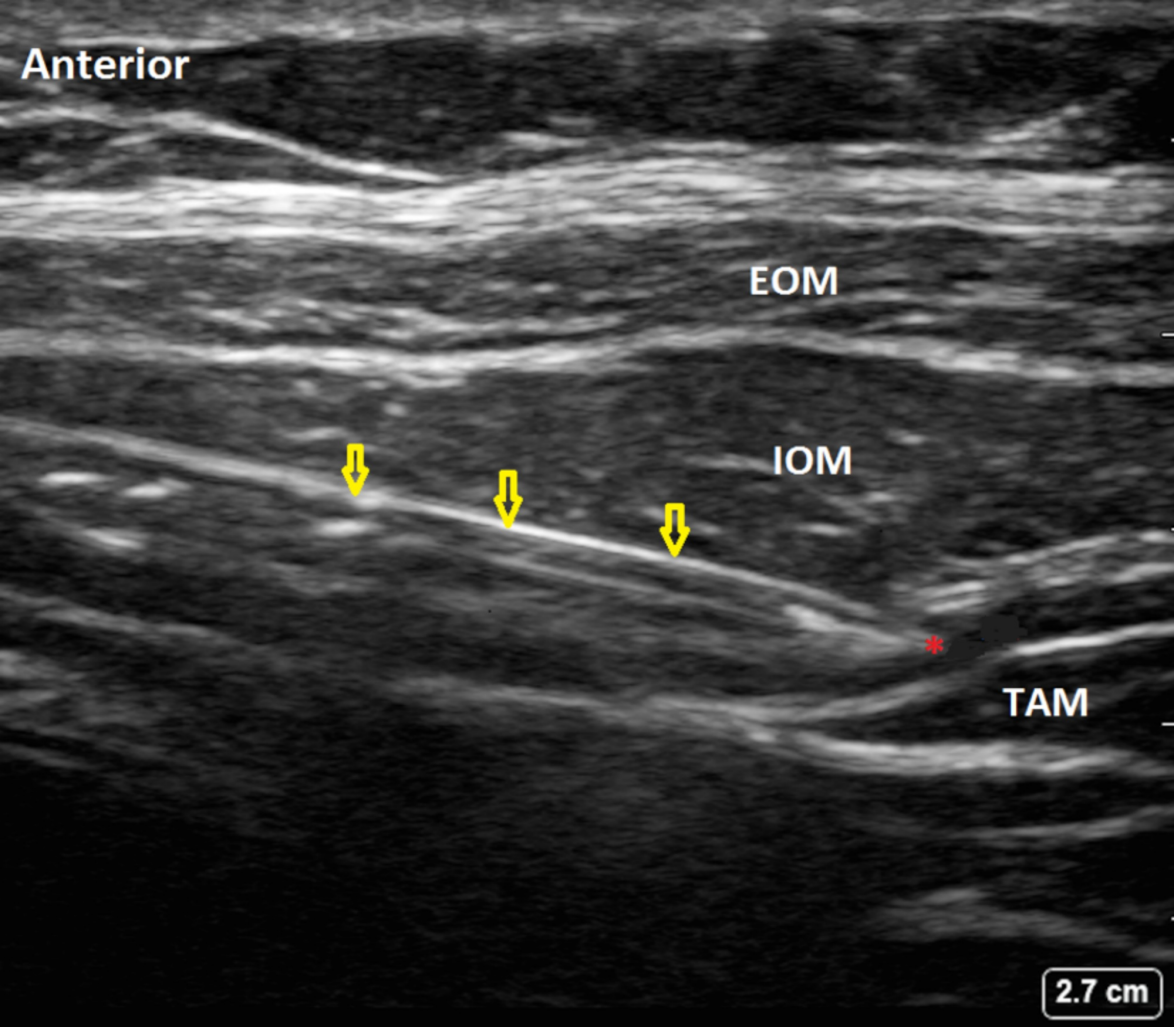
Ultrasound-guided Transversus Abdominis Plane (TAP) Injection. The yellow arrows indicate the needle, and the red (*) indicates the injection site. EOM: External oblique muscle; IOM: Internal oblique muscle; TAM: Transversus abdominis muscle.

We placed bilateral ultrasound-guided TAP catheters in a fresh cadaver without a history or anatomical evidence of prior abdominal surgery. In the supine position, a linear ultrasound probe (15-6 MHz) was guided over the abdomen at the mid-axillary line between the lower costal margins and iliac crest, and the lateral wall of the transversus abdominis fascial plane was visualized. Under ultrasound guidance, the TAP was identified with the injection of 5 cc of normal saline in the fascial plane between the internal oblique and transversus abdominis muscles (Figure 1), and a 5 Fr catheter was inserted over an 18 Ga needle (SOLO-DEX Fascile® Continuous Peripheral Nerve Block, Solo-Dex Inc., Boulder, CO) and secured. This procedure was performed bilaterally in the same fashion. The two catheters were attached to a 100 cc solution bag filled with 10 cc of 300 mgl/ml iohexol dye (Omnipaque, GE Healthcare Ireland, Cork, Ireland), and diluted with normal saline (0.9% sodium chloride solution) to a 1:10 ratio (calculated osmolality of 323 mOsmol/L). The catheter on the right side was programmed for PIB with an automated 5 mL hourly bolus, and the left side was programmed for a CI at 5 mL/h (PIB-PCA, ambit, Summit Medical Products, Sandy, UT).

**Figure 2:**
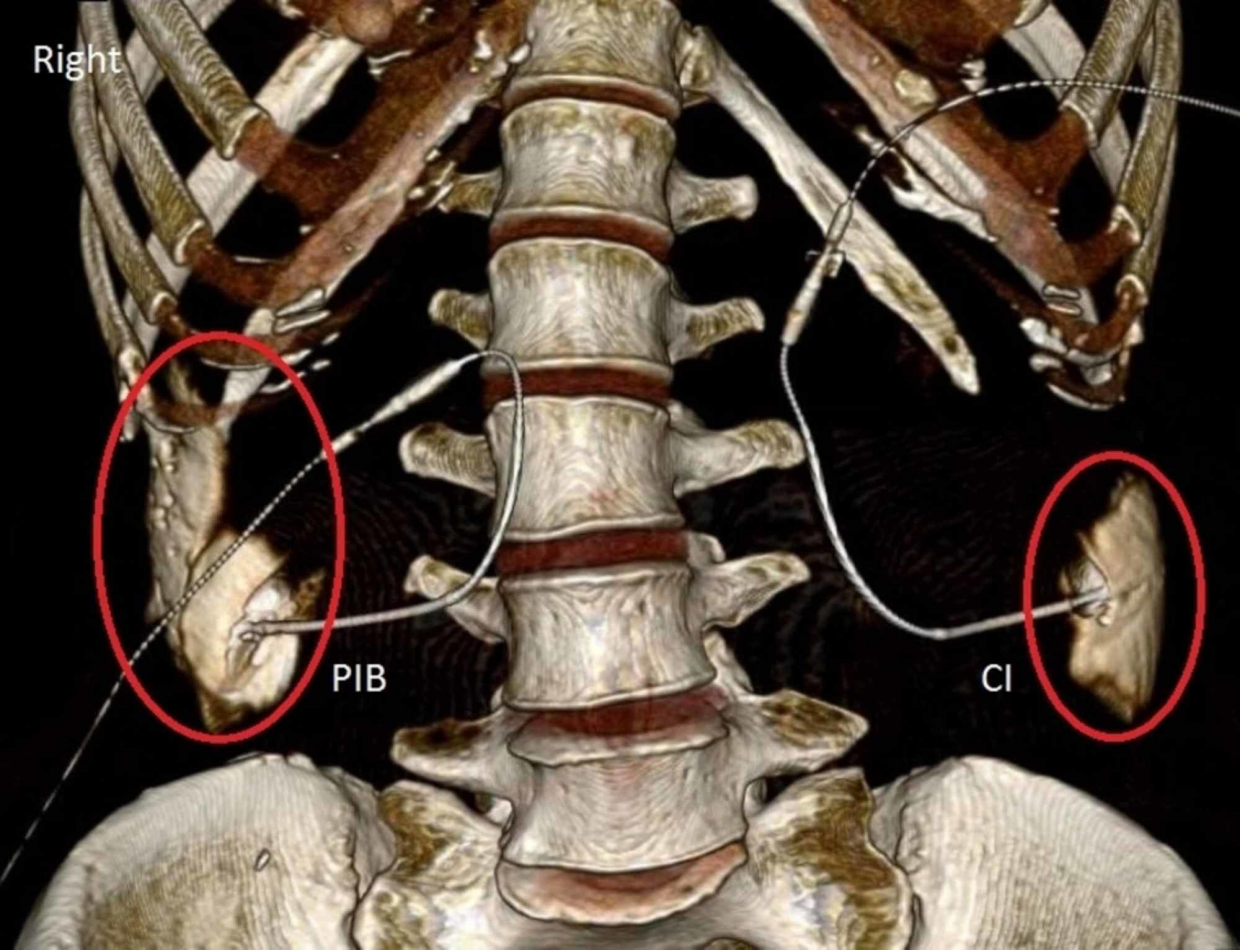
Distribution of Contrast after a Two-Hour Infusion. Labeled in red, is the spread of contrast in the transversus abdominis fascial plane. PIB: Programmed intermittent bolus; CI: Continuous infusion.

**Figure 3:**
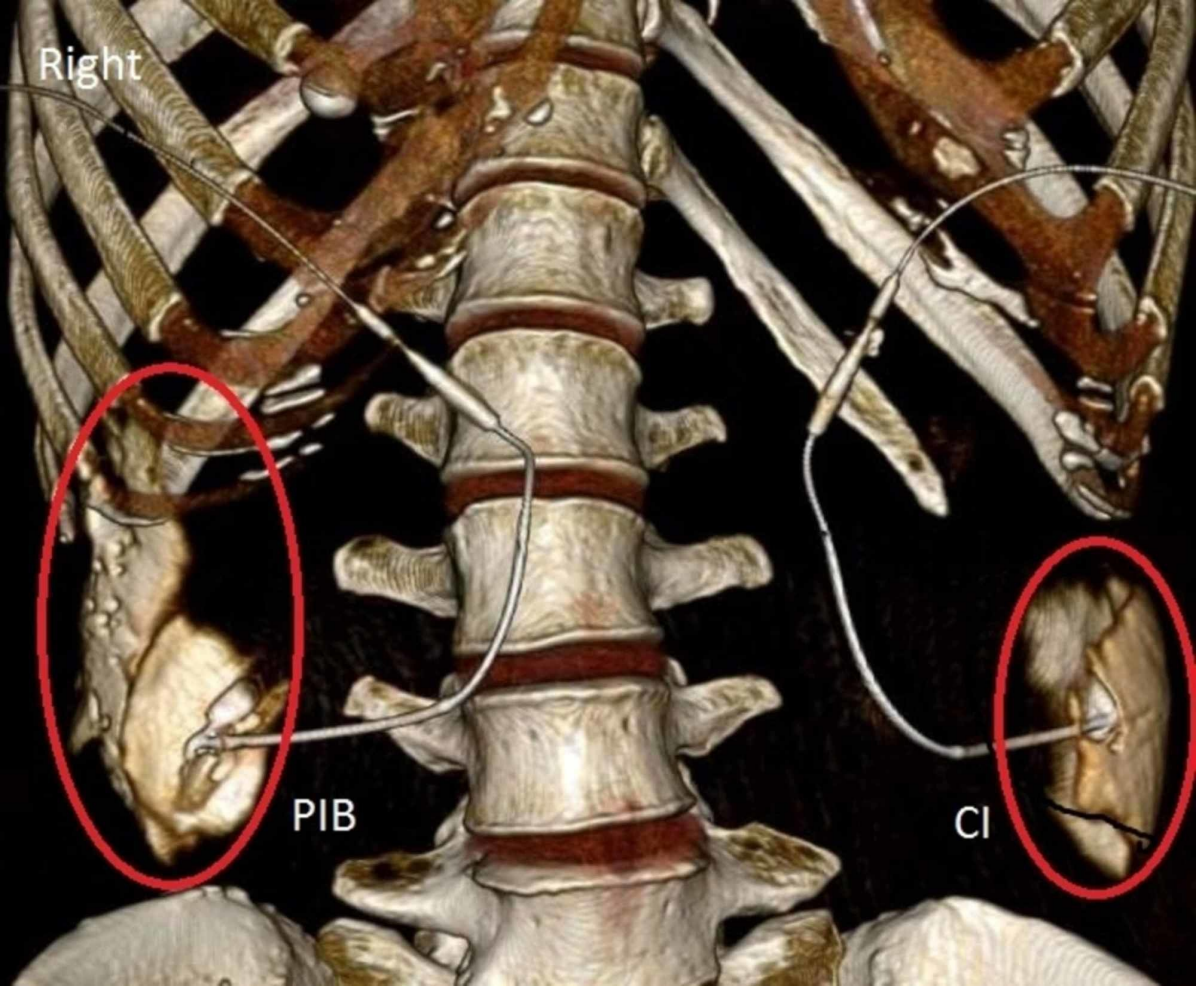
Distribution of Contrast after a Three-Hour Infusion. Labeled in red, is the spread of contrast in the transversus abdominis fascial plane. PIB: Programmed intermittent bolus; CI: Continuous infusion.

**Figure 4:**
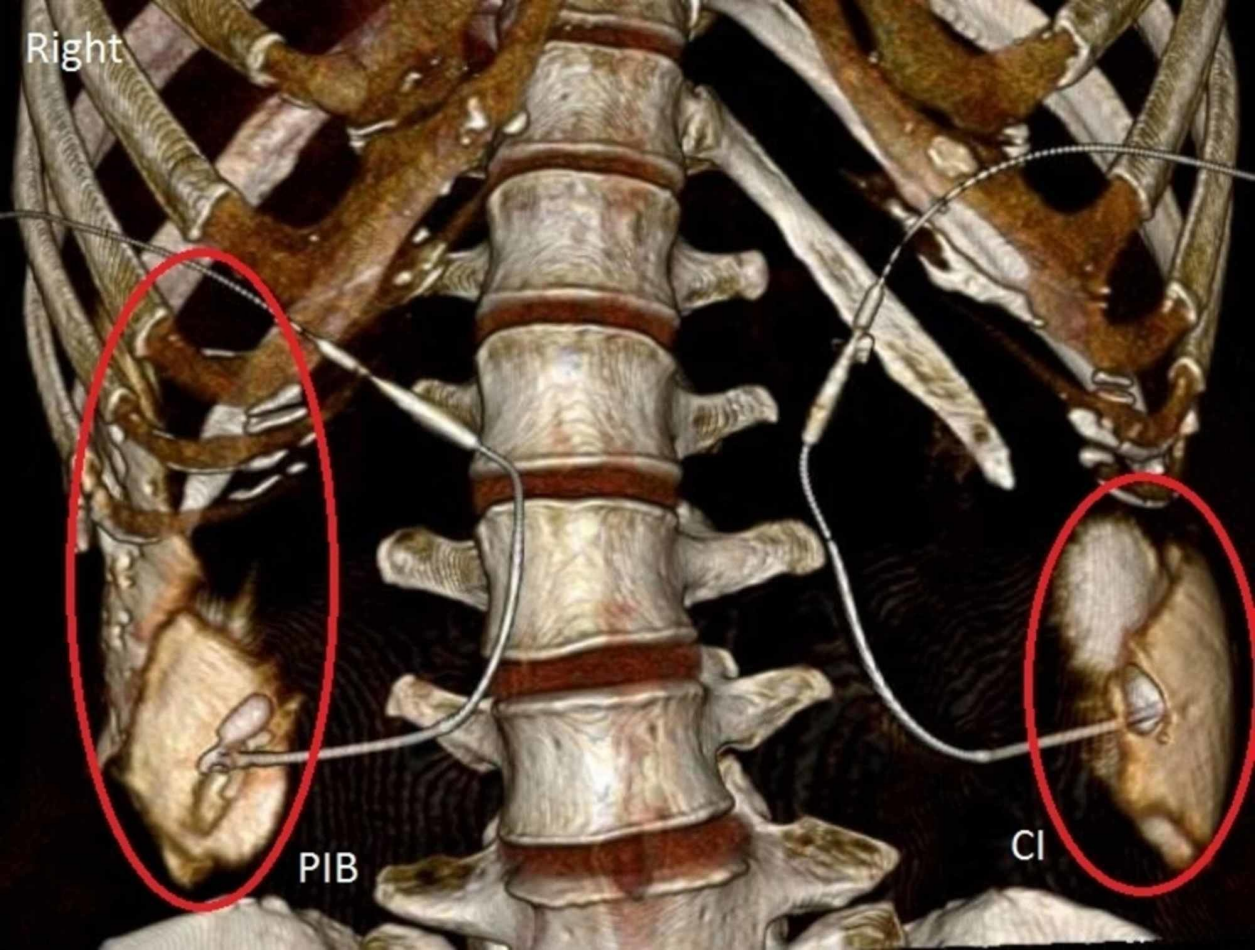
Distribution of Contrast after a Four-Hour Infusion. Labeled in red, is the spread of contrast in the transversus abdominis fascial plane. PIB: Programmed intermittent bolus; CI: Continuous infusion.

We transported and placed the cadaver in the supine position on a computed tomography (CT) scanner table and without any further movement, the infusions began. Volume rendering CT scans at 0.5 mm slices were obtained at three separate time intervals after the start of the infusion: two, three, and four hours. The CT imaging of the injection after two hours (Figure 2), three hours (Figure 3), and four hours (Figure 4) demonstrated that PIB mode of delivery, compared to CI, resulted in a greater spread of the injection with contrast traveling cephalad to the subcostal region.

## Discussion

The study suggests that delivery of medication in the transversus abdominis fascial plane via PIB results in greater anatomical coverage up to the subcostal area, and the most likely mechanism for this finding is the increased hydraulic pressure along the fascial plane created by the force of the bolus. Since the TAP block has been described to be limited in its ability to anesthetize the entire abdominal wall from T6 to L2, pulse dosing via a pump and through a catheter may improve efficacy. The TAP block is most commonly performed under ultrasound guidance by a lateral mid-axillary approach. Analgesia has been noted to be reliably produced from the T10 through L1 dermatomes due to the limited spread of injectate along the fascial plane [5]. To address this limitation, a subcostal variation of the TAP block was described by Hebbard et al., with the intent to cover the upper abdomen (T6 to T10) [6]. These techniques can then be combined, described by Borglum et al. as the dual TAP, in order to anesthetize the entire abdominal wall [7]. Therefore, for bilateral coverage of the abdominal wall, four injections of local anesthetics would be necessary using potentially four catheters. This approach would raise concerns with respect to increased doses of local anesthetic after four injections, and the logistical and economical barriers of placing and maintaining four catheters connected to four pumps. Based on our results, a continuous TAP block with the infusion programmed as PIB may combine the analgesic efficacy of the lateral and subcostal TAP. The likely mechanism for the radiological finding is the high rate of the injection at the time of delivery results in increased hydraulic pressure along the fascial plane created by the force of the bolus [8]. A CI set at 5 mL/hr infuses at 1 mL every 12 minutes; meanwhile, PIB set at 5 ml/hr to be administered at the end of each hour interval will deliver the dose at 210 ml/hr (as per manufactures specifications), thus 5 ml is infused in 86 seconds.

Currently, the evidence describing the clinical significance to our findings is limited. A randomized controlled trial on 20 patients conducted by Rao et al. on TAP block catheters placed after an open abdominal surgery with a midline incision (randomized to continuous infusion or intermittent bolus) found no significant difference in post-operative pain and opioid use [9]. However, all patients participating in the study had relatively low pain scores and received high doses of fentanyl throughout the investigation period. The details of the dermatomal coverage were brief with the bolus group described as covering T6 to L1 and the continuous group covering T6 to T12. A detailed report of the dermatomal coverage of the study participants would be of interest, since such broad coverage from a TAP block is inconsistent with what is typically observed in clinical practice and described in the literature [5-7]. A larger study on 120 patients receiving TAP block catheters for gastrointestinal surgery (randomized to continuous infusion or intermittent bolus), by Holmes et al., did not find a significant difference in analgesic outcome between the two modes of delivery either [10]. However, the study had a great deal of heterogeneity with potential confounders, such as different hospitals’ practices, variations in surgical complexity, and technical differences in the placement of the TAP catheters (ultrasound guided by an anesthesiologist or surgical placement with closure of the incision).

A limitation to our results is that the spread of the injection in a cadaver may not be the same as in a patient, and results may also differ between individuals. An imaging study in healthy volunteers with volume measurements would help confirm these results; and potential differences, such as osmolality, between a contrast dye solution and local anesthetics are important to consider. Future studies are necessary to investigate the clinical implications of PIB for TAP catheters with an emphasis on the analgesic profile of the block and dermatomal coverage.

## Conclusions

Based on cadaveric evidence, the PIB mode of infusion of a TAP catheter, as compared to CI, results in greater spread of the injection in the fascial plane and provides subcostal coverage. Clinical trials are needed to further investigate if these findings translate to the clinical setting.
